# A Dilated Pore of Winer on the Thigh: Clinical and Histopathological Correlation

**DOI:** 10.7759/cureus.95197

**Published:** 2025-10-22

**Authors:** Adnan Ahmad, Alsadat Mosbeh, Yashpal Manchanda, Kawthar Safar, Danah Al-Rasheedi, Fai Al-Saleeli, Abeer Albazali

**Affiliations:** 1 Dermatology, Farwaniya Hospital, Farwaniya, KWT; 2 Dermatology, Kuwait Institute for Medical Specializations, Sulibekhat, KWT; 3 Dermatology and Dermatopathology, Al-Azhar University, Cairo, EGY; 4 Dermatology, Amiri Hospital, Kuwait City, KWT; 5 Dermatology, Jahra Hospital, Jahra, KWT

**Keywords:** adnexal neoplasm, benign follicular tumor, dermatology, dermatopathology, dilated pore of winer, skin neoplasm

## Abstract

Dilated pore of Winer (DPW) is a benign follicular adnexal tumor that typically occurs in middle-aged and elderly individuals, most commonly on the face and neck. Rarely, it can present at unusual sites or in younger patients, leading to diagnostic challenges. We present the case of a 28-year-old female with a solitary, asymptomatic, comedo-like lesion on the right thigh that had been slowly enlarging over two years. Histological evaluation following surgical excision demonstrated a dilated follicular infundibulum filled with keratin and lined by acanthotic epithelium with radiating strands, consistent with DPW. The lesion was completely excised without any complications. This report expands the clinical spectrum of DPW by illustrating its occurrence in a young adult at an uncommon site and highlights the importance of histopathology in confirming the diagnosis.

## Introduction

Dilated pore of Winer (DPW), first described by Louis H. Winer in 1954, is a benign follicular adnexal tumor characterized clinically by a solitary, keratin-filled, comedo-like opening, and histologically by a markedly dilated follicular infundibulum with acanthotic epithelium and keratin plugging [1]. Later reviews, including Steffen’s analysis in 2001, confirmed DPW as a distinct follicular neoplasm with differentiation toward the infundibulum, separating it from ruptured cysts and other adnexal tumors [2]. Histological variants such as dilated pore nevus and aggregated dilated pores have been described, and unusual localizations such as the vulva have also been reported [3-5]. We report a rare case of DPW in a young woman involving the thigh, highlighting an atypical age and anatomical site.

## Case presentation

**Figure 1:**
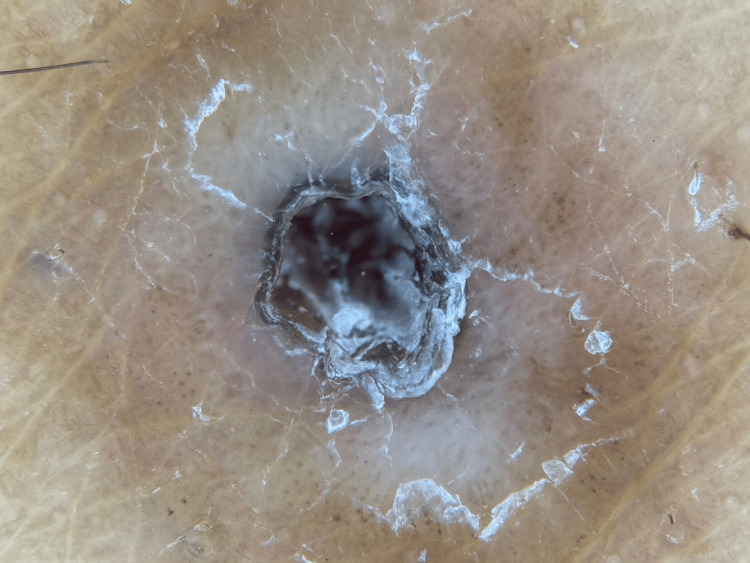
A solitary pore filled with keratin was observed on dermoscopy of the right thigh

A 28-year-old female presented with a two-year history of a slowly enlarging, asymptomatic, comedo-like swelling on the right thigh. The lesion measured approximately 0.5 cm × 0.5 cm × 0.6 cm and was occasionally associated with black, dry, odorless discharge (Figure 1). She denied significant past medical or surgical history, medication use, or drug allergies. Her concern was primarily cosmetic. Our differential diagnosis included epidermal inclusion cyst, trichofolliculoma, pilar sheath acanthoma, and nevus comedonicus.

**Figure 2:**
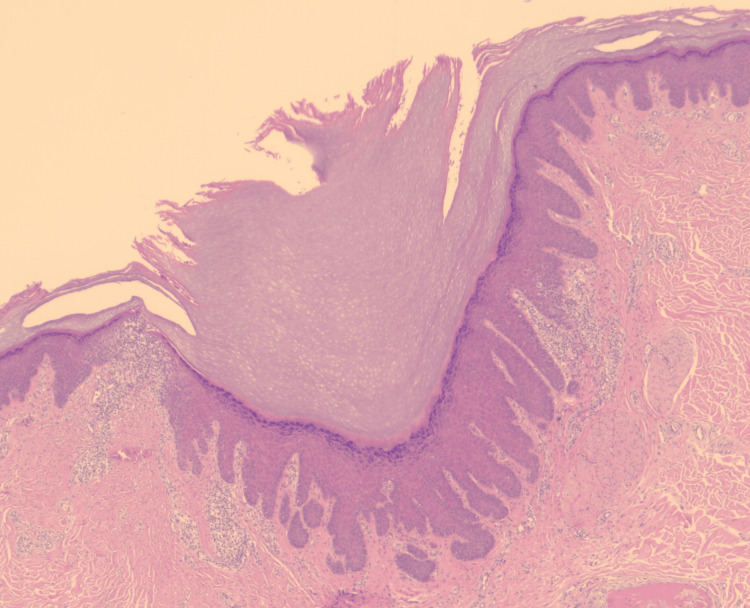
Microscopic examination showing a dilated follicular epithelium filled with keratin and radiating epithelial strands at the base (H&E, ×2.5) H&E: Hematoxillin and Eosin

The lesion was excised under local anesthesia and sent for histopathological evaluation. Using Hematoxillin and Eosin (H&E) stain, microscopic examination revealed a dilated follicular infundibulum filled with lamellar keratin. The epithelium was acanthotic, with radiating strands at the base and sides of the pore, and accompanied by a superficial perivascular lymphohistiocytic infiltrate (Figure 2). These findings confirmed the diagnosis of DPW.

## Discussion

DPW is an uncommon adnexal tumor of follicular origin. Winer first described the lesion in 1954, characterizing it by a keratin-filled ostium and histological features of downward epithelial proliferation with keratin plugging [1]. Later analyses, including Steffen’s detailed review in 2001, reinforced DPW as a distinct follicular neoplasm with differentiation toward the infundibulum, differentiating it from ruptured cysts and other adnexal tumors [2]. Histologic variants such as dilated pore nevus and aggregated dilated pores have been described [3,4]. Rare reports have also noted multiple or vulvar lesions, further expanding its clinical spectrum [5].

Our case is distinctive in that the lesion occurred in a 28-year-old patient and involved the thigh, thereby broadening the known clinical presentation of DPW. Other reports, such as Mittal et al.’s case in an Indian patient, also illustrate its occurrence outside the classical demographic and typical anatomical sites [6]. Rare associations have been documented, including DPW arising in conjunction with trichoblastoma, or even undergoing malignant transformation into squamous cell carcinoma [7,8].

The differential diagnosis for a keratin-filled lesion includes epidermal inclusion cyst, pilar cyst, comedone, nevus comedonicus, and trichofolliculoma. Epidermal cysts typically present with a central punctum but lack the radiating epithelial strands seen in DPW. Pilar cysts are usually located on the scalp and show trichilemmal keratinization. Comedones and nevus comedonicus may mimic DPW clinically, but histologically, they reveal multiple dilated follicular structures rather than a solitary, expanded pore [9]. Trichofolliculoma demonstrates secondary follicles budding from the infundibulum, a feature absent in DPW [10]. Careful histopathological assessment, therefore, remains essential for accurate diagnosis [9,10].

Histologically, DPW is characterized by a markedly dilated follicular infundibulum lined by atrophic epithelium at the surface and acanthotic epithelium at deeper levels, often producing radiating epithelial projections. The lumen is filled with lamellar keratin, features that were clearly observed in our patient. These findings are consistent with prior descriptions in the dermatopathology literature [9,10].

The present case underscores two unusual aspects: the early age of onset in a young adult and the atypical site of occurrence on the thigh. Recognition of such variants is important to avoid misdiagnosis with epidermal cysts, comedones, or adnexal tumors. Reporting rare presentations such as this contributes to awareness of the broader clinical spectrum of DPW and supports accurate diagnosis and appropriate management.

## Conclusions

DPW is a benign follicular neoplasm that classically presents in older adults on the head and neck. Our case highlights its occurrence in a young adult at an unusual site, reinforcing the need for histopathological confirmation. Surgical excision is curative, with excellent outcomes. Awareness of such rare presentations enhances diagnostic precision and broadens the clinical understanding of DPW.
